# Antibacterial and Antioxidant Potency of Floral Honeys from Different Botanical and Geographical Origins

**DOI:** 10.3390/molecules170910540

**Published:** 2012-09-04

**Authors:** Hasan A. Alzahrani, Rashid Alsabehi, Laïd Boukraâ, Fatiha Abde-llah, Yuva Bellik, Balkees A. Bakhotmah

**Affiliations:** 1 Mohammad Hussein Al Amoudi Chair for Diabetic Foot Research, King Abdulaziz University, Jeddah 21589, Saudi Arabia; Email: haaz59@yahoo.com (H.A.A.); alsabehi@hotmail.com (R.A.); hbaab1961@yahoo.com (B.A.B.); 2 Department of Surgery, Faculty of Medicine, King Abdulaziz University, Jeddah 21589, Saudi Arabia; 3 Department of Biology, Faculty of Sciences, King Abdulaziz University, Jeddah 21589, Saudi Arabia; 4 Laboratory of Research on Local Animal Products, Ibn-Khaldoun University of Tiaret, Tiaret 14000, Algeria; Email: fatiha.abdellah@yahoo.fr (F.A.); bellik_yuva@yahoo.fr (Y.B.); 5 Department of Nutrition Food Sciences, Arts and Design College, King Abdulaziz University, Jeddah 21589, Saudi Arabia

**Keywords:** honey, antibacterial, antioxidant

## Abstract

In order to assess their physicochemical and antioxidant properties as well as their antimicrobial potency, four varieties of honey from different botanical and geographical origins were used. The agar incorporation method was used to determine the antimicrobial potency of honeys. The total phenol content was determined by a modified Folin–Ciocalteu method and the free radical scavenging activity by the Fe^3+^ reducing power (FRAP) assay. Manuka honey was the most effective against *Staphylococcus aureus* Oxa R and *S. aureus* Oxa S with a Minimum Inhibitory Concentration (MIC) of 6% and 7%, respectively, whereas wild carrot honey was the most effective against *Pseudomonas aeruginosa*, with a MIC of 12%. Lavender honey was the least effective against all tested strains, even though was found to have the lowest pH and water content. Manuka honey had the highest content of polyphenols, with 899.09 ± 11.75 mg gallic acid/kg, whereas lavender honey had the lowest, with 111.42 ± 3.54 mg gallic acid/kg. A very significant correlation (*r* value was 0.9079 at *P* < 0.05) was observed between the total polyphenolic content and the Fe^2+^ content formed in the presence of the honey antioxidants. The differences between honey samples in terms of antibacterial and antioxidant activity could be attributed to the natural variations in floral sources of nectar and the different locations.

## 1. Introduction

It has been demonstrated in many studies that honey has antibacterial effects, attributed to its high osmolarity, low pH, hydrogen peroxide content, and content of other, uncharacterized compounds [1,2]. The low water activity of honey is inhibitory to the growth of the majority of bacteria, but this is not the only explanation for its antimicrobial activity. Molan [3] has studied sugar syrups of the same water activity as honey and found them to be less effective than honey at inhibiting microbial growth *in vitro*. Honey is mildly acidic, with a pH between 3.2 and 4.5. The low pH alone is inhibitory to many pathogenic bacteria and, in topical applications at least, could be sufficient to exert an inhibitory effect. When consumed orally, the honey would be so diluted by body fluids that any effect of low pH is likely to be lost [1]. Hydrogen peroxide was identified as the major source of antibacterial activity in honey [4]. It is produced by the action of glucose oxidase on glucose, producing gluconic acid. This is inhibited by excessive heat and low water activity [4]. The hydrogen peroxide concentration produced in honey activated by dilution is typically about 1,000 times less than in the 3% solution commonly used as an antiseptic [3]. More recently, a correlation has been established between the level of H_2_O_2_ and the degree of antimicrobial activity of honey. It was also suggested that H_2_O_2_ alone may not be sufficient to account for the antimicrobial activity [5]. There are a range of other, largely uncharacterized, substances present in some honeys that have antibacterial effects [6]. For example, manuka honey from New Zealand with nonperoxidal antibacterial activity has been found to be effective at a low concentration [7]. Antibacterial aromatic acids [8] and 10-HDA, the main royal jelly acid with antibacterial properties [9] have also been found in honey, as well as defensin-1 [10]. The strong antibacterial activity of manuka honey is due to the presence of the antibacterial substance methylglyoxal [11]. The antifungal activity of honey against *Candida albicans* has been reported in many studies [12,13,14].

Phenolic compounds are amongst the most important groups of compounds occurring in plants, and are found to exhibit anticarcinogenic, anti-inflammatory, antiatherogenic, antithrombotic, immune-modulating and analgesic activities and which may exert these functions as antioxidants [15,16,17,18,19,20]. The phenolic acids are generally divided into two sub-classes: the substituted benzoic acids and cinnamic acids, whereas the flavonoids present in honey are categorized into three classes with similar structure: flavonols, flavones and flavanones. These contribute significantly to honey color, taste and flavor and have beneficial health effects [21]. The composition of honey, including its phenolic compounds, is variable, depending mainly on the floral source and also other external factors, including seasonal and environmental factors as well as processing [22]. Thus, with different compositions of active compounds in honey collected from different locations, differences in honey properties are to be expected. Diastase numbers (DN), hydroxymethylfurfural (HMF), proline and sucrose are usually used as indicators of the ripeness and quality of honeys [22,23,24]. As not all honeys are created equal in terms of antimicrobial and antioxidant activity because of differences in levels of peroxide production and non-peroxide factors, which vary by floral source and processing, a comparative study has been conducted to establish the antibacterial and antioxidant potency of four varieties of honey from different botanical and geographical origins. Given the huge number of reports on the antioxidant properties of honey, the later might be a novel antioxidant in the management of chronic diseases commonly associated with oxidative stress [25]. The objective of this study was to evaluate the antibacterial and the antioxidant properties of four varieties of honey from different botanical and geographical origin, as well as to find out the correlation between the total phenolic content and the antioxidant activity.

## 2. Results and Discussion

Table 1 summarizes the physicochemical values of the different varieties of honey. Lavender honey had the lowest pH and water content and the highest amount of sucrose. Manuka honey had the lowest values of diastase number and proline and the highest HMF one. Acacia honey and lavender honey showed the lowest content of HMF, whereas wild carrot honey had the highest amount of proline. 

**Table 1:** Values of physicochemical properties of the different varieties of honey.

Honey type	pH	Water content	DN	HMF	Proline	Sucrose %
				mg/kg	mg/kg	
Manuka	4.40	19.2	5	90.88	325,59	0.01
Acacia	5.40	17	10.9	6.08	768,86	ND
Lavender	3.80	15.6	8	6.96	541,83	4.22
Wild carrot	4.62	16.4	8.3	13.98	927,22	ND

Table 2 shows the phenolic content of honeys. Manuka honey had the highest phenolic content whereas lavender honey had the lowest and the reducing power of honeys is in correlation with their phenolic content. A very significant correlation was observed between the total polyphenolic content and the Fe^2+^ content formed in the presence of the honey antioxidants (*r* value was 0.9079 at *P* < 0.05). Similar findings were reported by Alvarez-Suaez *et al.* [18,19]. 

**Table 2:** Phenol content (mg gallic acid/kg) and FRAP values of tested honeys.

Honey	TPC	FRAP value
(100 mg/mL)	(mg gallic acid/Kg)	(ABS_700_)
**Manuka**	899.09 ± 11.75	1.2106 ± 0.005
**Acacia**	627.56 ± 44.03	1.366 ± 0.06
**Lavender **	111.42 ± 3.54	0.2089 ± 0.022
**Wild carrot**	503.09 ± 8.29	0.6386 ± 0.05
**Trolox**	-	-
**BHT**	-	-

Table 3 shows the antimicrobial effectiveness of honeys against bacteria and *C. albicans*. All varieties exhibited potency against the tested strains. Bacteria were more susceptible to the action of honey than *C. albicans.* Manuka honey exhibited the highest overall potency.

**Table 3:** The antibacterial potency of honeys against the tested strains.

Honey variety	MIC% of the four varieties against the tested microbes			
	*S. aureus* ATCC 43300 (Oxa R)	*S. aureus* ATCC 25923 (Oxa S)	*P. aeruginosa* ATCC27853	*Candida albicans*
Manuka	7	6	14	30
Acacia	10	12	13	32
Lavender	25	25	21	40
Wild carrot	11	13	12	36

The Codex Alimentarius [26] has established the minimum diastase activity value of 3 for honeys with natural low enzyme content. In honeys with a DN less than 8 and higher than or equal to 3, the HMF must not be higher than 15 mg/kg. If DN is equal to or higher than 8, HMF limit is 60 mg/kg. The HMF measurement is used to evaluate the quality of honey; generally not present in fresh honey, its content increases during conditioning and storage [27]. This seems to be the case of manuka honey in our experiment which has a lower value of DN and a higher value of HMF than the norms. The Codex Alimentarius (Alinorm 01/25 2000) has established that the HMF content of honey after processing and/or blending must not be higher than 80 mg/kg. The European Union (EU Directive 110/2001), however, recommends a lower limit of 40 mg/kg with the following exceptions: 80 mg/kg is allowed for honey that originates from countries or regions with tropical temperatures, while a lower limit of only 15 mg/kg is allowed for honey with low enzymatic levels [28]. Except manuka honey, all honeys in this study meet the standards for DN and HMF. The concentration of proline also serves as an additional determinant of quality and as a criterion for estimating the maturity of honey as well as an indicator for detecting sugar adulteration [29]. The minimum value of proline in honey allowed by Food Codex and Council of the European Union (CEU) must be above 180 mg /kg honey [30]. All studied honeys are above this limit of proline, with variety 4 showing the highest value. Sucrose content is important to detect heavy sugar feeding of the bees or adulteration by direct addition of saccharose. According to some studies, the amount of sucrose has been used to discriminate the adulteration of honey samples by sugar syrups. For example, supplementary feeding of honey bees with saccharose syrup caused a higher saccharose level in honey [31]. This seems to be the case of lavender honey, which exhibited a higher sugar content. Regarding the antimicrobial activity of honey, variety 1 (manuka) has showed the highest overall effectiveness even it was the least in terms of physicochemical properties. This high potency of manuka honey against bacteria was reported by Willix *et al.* [32], Molan [33] and Bogdanov [34]. Recently, Henriques *et al*. discussed the effect of manuka honey on the structural changes in *P. aeruginosa* [35]. The effect of manuka honey on *S. aureus* was also reported [36]. This is most probably due to its phenolic content. Methylglyoxyal (MGO) has been identified as the dominant bioactive component in manuka honey (*Leptospermum scoparium*) and its concentration was correlated to the non-peroxide activity of honey [11,37]. However, Jervis-Bardy *et al.* reported that MGO is only partially responsible for the antibacterial activity of manuka honey [38]. Davidson *et al.* [39] have shown that individual phenolic compounds have growth inhibition on a wide range of Gram-positive and Gram-negative bacteria. Alvarez-Suarez *et al.* reported that *S. aureus* is more sensitive to the action of honey that *P. aeruginosa*, [40] which is in agreement with our findings. In a recent study, it was demonstrated that honey H_2_O_2_ exerted bacteriostatic and DNA degrading effects on bacterial cells. This is strongly influenced by the bacterial sensitivity to oxidative stress [41]. Later it was demonstrated that bacteriostatic effect of honeys was related to generation of •OH from honey H_2_O_2_ in a dose dependent manner [42]. All honeys were effective against *Candida albicans*. The antifungal action of honey has been observed in previous studies for some yeast, such as *C. albicans*, and species of *Aspergillus* and *Penicillium*, as well as all the common dermatophytes [12,13,14,43]. Lavender honey, which shows the least overall antimicrobial activity and likely related to its content in polyphenols, is widely used topically by urban Saudis to treat foot ulcers [44]. Among the compounds found in honey; vitamin C, phenol compounds, catalase, peroxides, glucose oxidase enzymes have antioxidant properties [45,46]. Honey also contains flavonoids and carotinoids [47,48]. High levels of these indicators ensure a high level of antioxidants in honey. Research showed that darker honeys provide highest levels of antioxidants as well as antimicrobial effectiveness.

## 3. Experimental

### 3.1. Honey Samples

Manuka honey (V1: Leptospermum *scoparium*) was purchased from Medihoney^®^ in the UK, black forest honey (V2: *Acacia*) was produced by Langanese Honig Germany, lavender honey (V3: *Lavender*) was provided by a beekeeper from Southern Saudi Arabia and wild carrot honey (V4: *Daucus carota* L) was obtained from an Algerian beekeeper. 

### 3.2. Physicochemical Analysis

Moisture in honey was determined in a refractometer (Jena 181282, Carl Zeiss, Oberkochen, Germany), and the pH of the honey solution was measured by a pH meter (CG840 Schott, Gerate GmbH, Hamburg, Germany). Diastase activity was determined by the method of Horwitz [49] where the diastase activity is expressed as mL of 1% starch solution hydrolyzed by the enzyme in 1 g of honey during 1 h. HMF content was measured according to the method of White [24] and was based on the determination of UV absorbance of HMF at 284 nm. The results are expressed in milligrams per kilogram (mg/Kg). Sucrose and proline were determined according to an AOAC method [50].

### 3.3. Bacterial Analysis

#### 3.3.1. Bacterial Strains and Inoculum Standardization

*Staphylococcus* aureus 43300 (Oxa R), *Staphylococcus aureus* 25923 (Oxa S) and *Pseudomonas aeruginosa* ATCC 27853 were kindly provided by the university hospital of Algiers (Algeria). *Candida albicans* was graciously provided by “l’Institut Pasteur d’Alger” (Algeria). Prior to the experiment the strains were inoculated onto the surface of nutrient agar media; the inoculum suspensions were obtained by taking five colonies from 24 h cultures. The colonies were suspended in 5 mL of sterile saline (0.85% NaCl) and shaken for 15 seconds. The density was adjusted to the turbidity of a 0.5 McFarland Standard (equivalent to 1–5 × 10^8^ cfu/mL).

#### 3.3.2. Minimum Inhibitory Concentration Measurement (MIC)

Using the incorporation method, concentrations of honey between 5% and 30% (vol/vol) were added into Mueller Hinton agar media to test their efficiency against bacteria. For *Candida albicans*, concentrations of honey between 20% and 40% were incorporated into Sabouraud agar media. The final volume of honey and media in each plate (60 mm) was 5 mL. The plates were inoculated and incubated at 37 °C for 24 h for bacteria and at 35 °C for 48 h for *C. albicans*. The minimum inhibitory concentration (MIC) was determined by finding the plates with the lowest concentration of honey on which the strain would not grow. Tests were repeated in triplicate. All MIC values are expressed in percentage (vol/vol).

### 3.4. Antioxidant Activity

#### 3.4.1. Total Phenol Contents (TPC)

The total phenol content was determined by a modification of the Folin-Ciocalteu method as described by Beretta *et al.* [51]. One g of honey was treated with distilled water (10 mL), mixed and filtered using a qualitative filter (a No. 40 filter paper (Whatman, Cambridge, England). An aliquot of this solution (200 µL) was mixed with Folin-Ciocalteu reagent (500 µL, 10%) for 5 min and then a Na_2_CO_3_ solution were added (1,500 µL, 7.5%). All samples were incubated at room temperature in the dark conditions for 30 min, and their absorbance was read at 765 nm. Total phenolic content was expressed as mg gallic acid equivalents (GAE)/kg of honey from a calibration curve using the equation: y = 0.0094 xs + 0.0299 (R^2^ = 0.998). All samples were analyzed in triplicate.

#### 3.4.2. FRAP Assay

The FRAP assay is one of the most frequently used analytical strategies for antioxidant activity. As for Total Phenol Content assay, it consists in evaluating the level of polyphenols in sample. Several works had been reported that the antioxidant activity of many compounds of botanical origin is proportional to the phenolic content, suggesting a causative relationship between total phenolic content and antioxidant activity [52]. The Fe^3+^ reducing power of honey was determined by the method of Yen and Duh [53] with slight modifications. Honey (2.5 mL) was mixed with phosphate buffer (2.5 mL, 0.2 M, pH 6.6) and 1% potassium ferricyanide (2.5 mL). The mixtures were incubated for 20 min at 50 °C. After incubation, 10% trichloroacetic acid (2.5 mL) was added to the mixtures, followed by centrifugation at 3000 rpm for 10 min. The upper layer (1 mL) was mixed with distilled water (1 mL) and 0.1% ferric chloride (0.5 mL). The absorbance of the obtained solution was measured at 700 nm.

## 4. Statistical Analysis

Correlations were established using Pearson’s correlation coefficient (*r*) in bivariate linear correlations (*P* < 0.01). These correlations were calculated using Microsoft office Excel 2007 and SPSS version 18.0 [54].

## 5. Conclusions

As it has been demonstrated in many studies, honey has a potent activity against bacteria and fungi. This potency is attributed to its physicochemical and phytochemical characteristics. Phenolic compounds play a major role in the antimicrobial activity of honey and the differences between honey samples in terms of antibacterial and antioxidant activity could be attributed to the natural variations in floral sources of nectar and the different locations. Although honey by itself may not serve as a major source of dietary antioxidants, it demonstrates the potential to play a role in providing antioxidants in a highly palatable form. Due to honey’s pleasing taste, it may be more readily consumed by individuals reluctant to ingest plant-derived antioxidants.
